# Despite early Medicaid expansion, decreased durable virologic suppression among publicly insured people with HIV in Washington, DC: a retrospective analysis

**DOI:** 10.1186/s12889-020-08631-7

**Published:** 2020-04-16

**Authors:** Deborah Goldstein, W. David Hardy, Anne Monroe, Qingjiang Hou, Rachel Hart, Arpi Terzian, on behalf of the DC Cohort Executive Committee, Thilakavathy Subramanian, Jeffery Binkley, Rob Taylor, Nabil Rayeed, Cheryl Akridge, Stacey Purinton, Jeff Naughton, Lawrence D’Angelo, Natella Rahkmanina, Michael Kharfen, Angela Wood, Michael Serlin, Princy Kumar, David Parenti, Amanda Castel, Alan Greenberg, Anne Monroe, Lindsey Powers Happ, Maria Jaurretche, Brittany Lewis, James Peterson, Naji Younes, Ronald Wilcox, Sohail Ranam Michael Horberg, Ricardo Fernandez, Carl Dieffenbach, Henry Masur, Jose Bordon, Gebeyehu Teferi, Debra Benator, Deborah Goldstein, David Hardy

**Affiliations:** 1 Whitman-Walker Institute, 1525 14th Street, NW, Washington, DC 20005 USA; 2 Division of Infectious Diseases, Department of Medicine, Georgetown University Medical Center, Washington, DC USA; 3 Division of Infectious Diseases, Johns Hopkins University School of Medicine, Baltimore, MD USA; 4 Department of Epidemiology and Biostatistics, Milken Institute School of Public Health, George Washington University, Washington, DC USA; 5 Cerner Corporation, Kansas City, MO USA; 6 Patient-centered Outcomes Research Institute, Washington, DC USA

**Keywords:** Insurance coverage, HIV, Disparities, Antiretroviral therapy, Medicaid

## Abstract

**Background:**

Despite widely available access to HIV care in Washington, DC, inequities in HIV outcomes persist. We hypothesized that laboratory monitoring and virologic outcomes would not differ significantly based on insurance type.

**Methods:**

We compared HIV monitoring with outcomes among people with HIV (PWH) with private (commercial payer) versus public (Medicare, Medicaid) insurance receiving care at community and hospital clinics. The DC Cohort follows over 8000 PWH from 14 clinics. We included those ≥18 years old enrolled between 2011 and 2015 with stable insurance. Outcomes included frequency of CD4 count and HIV RNA monitoring (> 2 lab measures/year, > 30 days apart) and durable viral suppression (VS; HIV RNA < 50 copies/mL at last visit and receiving antiretroviral therapy (ART) for ≥12 months). Multivariable logistic regression models examined impact of demographic and clinical factors.

**Results:**

Among 3908 PWH, 67.9% were publicly-insured and 58.9% attended community clinics. Compared with privately insured participants, a higher proportion of publicly insured participants had the following characteristics: female sex, Black race, heterosexual, unemployed, and attending community clinics. Despite less lab monitoring, privately-insured PWH had greater durable VS than publicly-insured PWH (ART-naïve: private 70.0% vs public 53.1%, *p* = 0.03; ART-experienced: private 80.2% vs public 69.4%, *p* < 0.0001). Privately-insured PWH had greater durable VS than publicly-insured PWH at hospital clinics (AOR = 1.59, 95% CI: 1.20, 2.12; *p* = 0.001).

**Conclusions:**

Paradoxical differences between HIV monitoring and durable VS exist among publicly and privately-insured PWH in Washington, DC. Programs serving PWH must improve efforts to address barriers creating inequity in HIV outcomes.

## Background

For people with HIV (PWH), health insurance coverage is associated with sustained viral suppression (VS), decreased incidence of AIDS, reduced hospitalization rates, and reduced mortality [1–4] . PWH in the US are disproportionately uninsured, underinsured, or have public insurance, according to data prior to full Affordable Care Act (ACA) implementation [5, 6]. Prior to the ACA, approximately 17% of PWH were uninsured, and the majority received Ryan White assistance for HIV-related care [7]. Uninsured or publicly insured PWH have worse health outcomes compared to those who were privately-insured. Medicaid recipients initiated antiretroviral therapy (ART) at a more advanced stage of HIV disease than PWH who were privately-insured [8], and had a greater incidence of comorbidities including cardiovascular disease, renal impairment, and chronic hepatitis [2]. PWH with private insurance were more likely to have sustained VS compared with those with public insurance, according to prior studies [1, 5]. Additionally, PWH with public insurance in the HIV Outpatient Study had higher mortality rates than privately insured participants [2].

Health outcomes for low-income PWH in the District of Columbia (DC) may not mirror those of the rest of the country, however. DC’s AIDS Drug Assistance Program (ADAP) boasts an all-inclusive list of antiretrovirals (ARVs) and has never had a waiting list [9]. Since 2006, low-income residents ineligible for Medicaid or Medicare may enroll in the DC Healthcare Alliance, a healthcare safety-net program. The ACA was passed in 2010 with the aim of expanding Medicaid coverage to millions of low-income Americans. DC received a waiver from the Centers for Medicaid and Medicare Services to begin early Medicaid expansion in 2010. With eligibility levels among the most generous in the nation, DC Medicaid now covers nearly 40% of the District’s population [10]. While ADAP defrays the cost of HIV medications, Medicaid offers a broader array of services, including covering fees for medical and non-medical provider visits, non-HIV medications, laboratory testing, and referrals.

The DC Cohort, a district-wide, prospective, observational clinical cohort of PWH at 14 healthcare centers, has examined laboratory monitoring, insurance, clinic type, and HIV outcomes. Among Cohort participants, 84% were in continuous HIV care and 78 and 80% underwent regular CD4 and VL monitoring, respectively [11]. From 2011 to 2014, publicly-insured PWH were less likely to achieve VS; privately-insured PWH had an earlier time to VS; and public insurance was associated with earlier time to virologic failure [9]. Further, PWH concurrently receiving care at three or more clinics were more likely to have public insurance and detectable VL, as well as hypertension, cardiovascular disease, and mental health issues [12]. To further probe these findings, we first analyzed demographic characteristics of DC Cohort participants by insurance type. We then assessed the role of insurance type and clinic type on HIV outcomes within the DC Cohort to examine whether, in the setting of widely available health care coverage in Washington, DC, PWH with public insurance were more likely to have suboptimal HIV outcomes than PWH with private insurance. Finally, we analyzed the relationship between meeting laboratory monitoring standards and durable VS.

## Methods

### Study setting and population

The DC Cohort enrolls PWH from clinics in Washington, DC and merges their data into a centralized electronic record [13]. Participants’ clinical and billing data are abstracted from patient medical records and entered into a web-based data entry system called Discovere® (Cerner Corporation, Kansas City, MO). We included all active participants aged 18 years and above enrolled in the DC Cohort from 1/1/2011 through 9/30/2015 with at least 12 months of clinical visits or laboratory data prior to 9/30/2016. Patients who switched insurance after their DC Cohort enrollment and those whose insurance information was ‘other’ (clinical research study, Other, Insurance terminated, or Self pay) or ‘unknown’ were excluded.

Of 14 DC cohort clinics, 12 were included in this analysis: one clinic was excluded because participants had insufficient follow up time to be included; and the Veterans Administration (VA) Medical Center was excluded due to structural differences in the funding of medical care at this site. Of the 12 clinics, seven are based in private academic hospitals (‘hospital clinics’); four are federally qualified health centers and one is a private nonprofit community clinic (‘community clinics’) (DC Cohort Data and Statistics Coordinating Center. DC Cohort Site Specific Benchmark Report. March, 2015).

The study protocol, consent forms, and research instruments were approved by the George Washington University Institutional Review Board (IRB), the DC Department of Health (DOH) IRB, and the IRBs of the individual clinics. Details of the DC Cohort study design have been described previously [13].

### Main predictor

Eligible patients’ primary insurance type was classified as public or private; secondary insurance type was not analyzed. Public insurance included Medicare, Medicaid, Ryan White/ADAP, DC Alliance, or other form of public insurance. Private insurance included commercial payer policies and Affordable Care Act plans. No patients were classified as uninsured.

### Main outcomes

HIV outcomes were those defined by the Institute of Medicine (IOM) [14], specifically regular CD4 cell and HIV RNA monitoring and durable VS. Regular CD4 and VL monitoring were defined as ≥2 CD4 and ≥ 2 VL test results in the 12 months following the index date, with at least 30 days between tests. Index date was defined as the later of two dates: 1) date of ART initiation, and 2) date of enrollment into the DC Cohort. Durable VS was defined as HIV RNA < 50 copies/mL at last lab assay among PWH on ART for ≥12 months.

### Treatment initiation type

ART status was determined at enrollment date. Participants with a reported history of taking ARVs before enrollment were classified as treatment-experienced; participants with no reported history of taking ARVs before enrollment were classified as treatment-naïve. Treatment initiation is a proxy for duration of infection and previous exposure to ARVs before enrollment.

### Data analysis

Descriptive statistics were generated. Differences by ART status were assessed using χ^2^ or two sample t-tests, as appropriate.

To assess the main effect of insurance type on durable VS among both ART-naïve and ART-experienced participants, a multivariable logistic regression model was computed. Models were adjusted for demographic characteristics (age, gender, race/ethnicity, housing and employment status), years since HIV diagnosis, HIV transmission risk categories, and AIDS diagnosis. Sum of comorbid diseases (drug abuse, depression, psychotic disorder, hypertension, and hepatitis C) was calculated. The covariate age was considered as both a continuous variable categorized in 10-year intervals and as a fractional polynomial to assess its linearity on outcomes. Linearity was confirmed and age as a continuous variable categorized in 10-year intervals was selected for inclusion in the final model. Interaction effects by both insurance and clinic site were also assessed.

Several multivariable models were computed, including backward elimination, stepwise and forward models. Model goodness of fit for each of these three modelling strategies was assessed using Hosmer-Lemeshow statistic with χ^2^ tests. Estimates were compared across models. The final model, the backward elimination model, was selected based on its model fit and parsimony relative to the other models. The five most common comorbid conditions (drug abuse, depression, psychotic disorder, hypertension, and hepatitis C) were modeled as the sum of all conditions to reflect additional burden of increasing comorbidities. The joint effect was not statistically significant on the relationship between insurance and outcomes and therefore it was excluded in the final model. All analyses were conducted in SAS^R^ (version 9.3), Copyright© 2011, SAS Institute Inc., Cary, NC, USA.

## Results

**Fig. 1:**
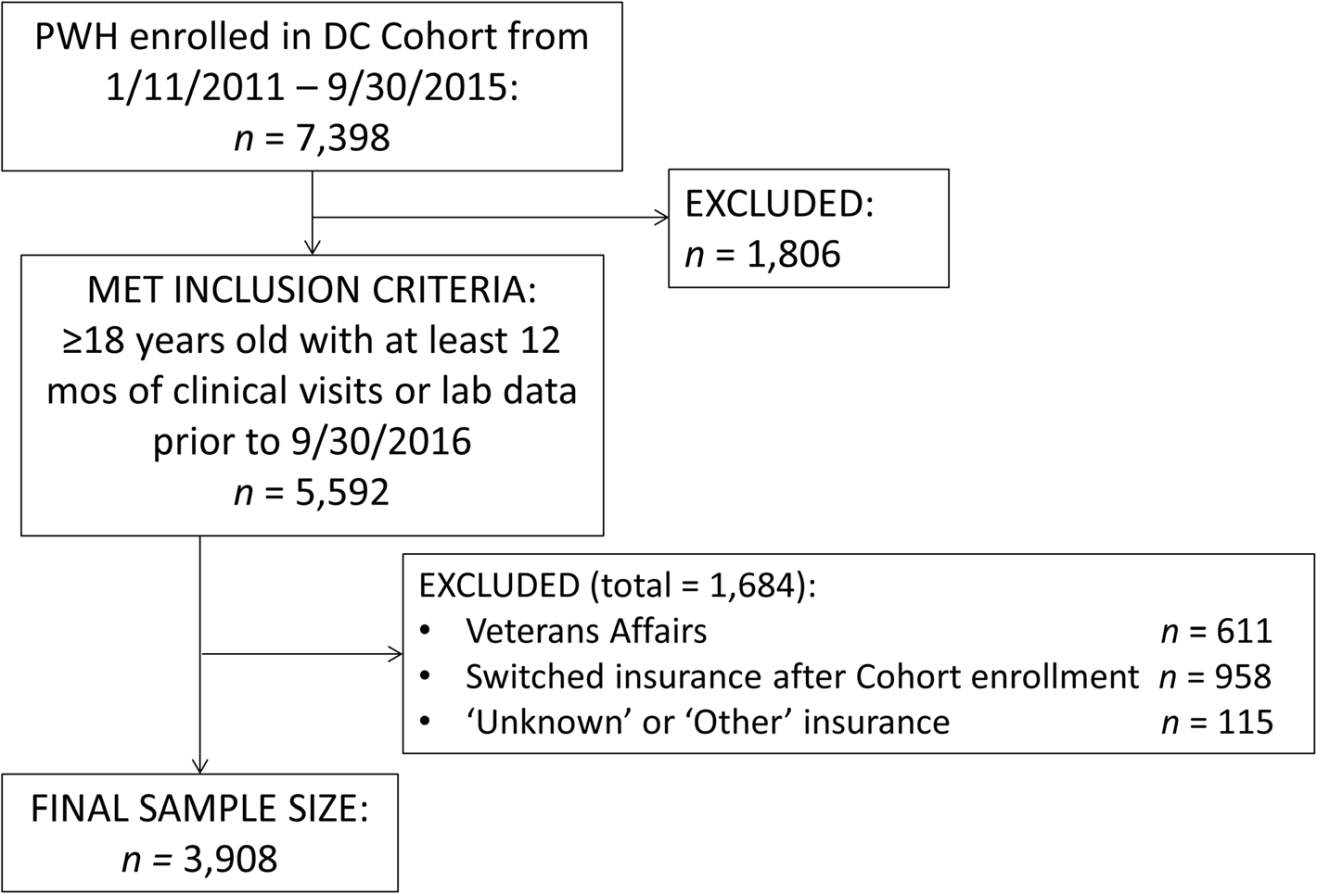
Study Attrition Flowchart

Of 7389 patients enrolled in the DC Cohort from 1/1/2011 through 9/30/2015, 3908 met inclusion criteria for this analysis (Fig. 1).

Of these 3908 PWH, 2652 (67.9%) were publicly-insured, 3723 (95.3%) were ART-experienced at study enrollment and 2300 (58.9%) received care at community clinics. In treatment-stratified comparisons by insurance status, publicly-insured PWH comprised 68.1% of the ART-experienced and 62.2% of the ART-naïve groups (see Table 1). Compared with privately insured PWH, a higher proportion of publicly-insured PWH were female, black, heterosexual, unemployed, and receiving care at community clinics. Overall, publicly-insured PWH had a higher prevalence of the five comorbidities assessed at enrollment than privately-insured PWH.

**Table 1:** Sociodemographic and clinical characteristics by antiretroviral therapy (ART) status among DC Cohort participants at study enrollment, including comparisons within ART status by insurance type in Washington, DC, 2011–2015

		ART-experienced		ART-naïve			ART-experienced Only^2^	ART-naïve Only^3^
		N (%) 3723 (95.3)		N (%) 185 (4.7)			Public vs Private	Public vs Private
Characteristic	Total N (%)	Public	Private	Public	Private	*P*-value^1^	*P*-value	*P*-value
	3908	2537(68.1)	1186(31.9)	115(62.2)	70(37.8)	0.089		
**Age**								
Mean	46.3	47.3	45.3	40.0	39.2	**<.0001**	**<.0001**	0.693
IQR	38.0–55.0	40.0–56.0	38.0–53.0	26.0–53.0	31.0–46.0			
**Years since HIV diagnosis**^**4**^								
Mean	13.3	13.4	14.0	7.3	5.9	**<.0001**	0.696	**0.043**
IQR	6.4–19.1	6.3–19.1	7.9–20.1	2.5–8.1	1.6–7.0			
**Sex at birth**								
Female	1190(30.4)	918(36.2)	227(19.1)	37(32.2)	8(11.4)	0.064	**<.0001**	**0.001**
Male	2718(69.6)	1619(63.8)	959(80.9)	78(67.8)	62(88.6)			
**Race/Ethnicity**								
Hispanic	198(4.8)	115(4.5)	61(5.1)	4(3.5)	9(12.9)	0.420	**<.0001**	**<.0001**
NH Black	3025(77.4)	2204(86.7)	678(57.2)	107(93.0)	36(51.4)			
NH White	538(13.8)	160(6.3)	354(29.9)	2(1.7)	22(31.4)			
Other/Unknown	156(4.0)	58(2.3)	93(7.8)	2(1.7)	3(4.3)			
**Housing**								
Permanent	3226 (82.6)	1959(77.2)	1125(94.9)	78(67.8)	64(91.4)	0.076	**<.0001**	**0.001**
Temporary	272(7.0)	245(9.7)	12(1.0)	14(12.2)	1(1.4)			
Other/Unknown	410(10.5)	333(13.1)	49(4.1)	23(20.0)	5(7.1)			
**Employment**								
Employed	1220(31.2)	336(13.2)	812(68.5)	23(20.0)	49(70.0)	0.076	**<.0001**	**<.0001**
Unemployed	1248(31.9)	1119(44.1)	76(6.4)	42(36.5)	3(4.3)			
Other/Unknown	1440(36.9)	1082(42.6)	298(25.1)	50(43.5)	18(25.7)			
**HIV Risk**								
MSM	1467(37.5)	716(28.2)	664(56.0)	39(33.9)	48(68.6)	**0.027**	**<.0001**	**<.0001**
IDU	242(6.2)	196(7.7)	33(2.8)	10(8.7)	3(4.3)			
Heterosexual	1325(33.9)	981(38.7)	289(24.4)	46(40.0)	9(12.3)			
Other/Unknown^5^	874(22.4)	644(25.4)	200(16.9)	20(17.4)	10(14.3)			
**History of AIDS**	1424(36.4)	938(36.9)	459(38.7)	13(11.3)	14(20.0)	**<.0001**	0.309	0.130
**Clinic site**								
Hospital	1608(41.2)	601(23.7)	912(76.9)	42(36.5)	53(75.7)	**0.005**	**<.0001**	**0.001**
Community	2300(58.9)	1936(76.3)	274 23.1)	73(63.5)	17(24.3)			
**Comorbidities**^**6**^								
Drug abuse	625(16.0)	539(21.3)	58(4.9)	23(20.0)	5(7.1)	0.837	**<.0001**	**0.020**
Depression	770(19.7)	542(21.4)	198(16.7)	22(19.1)	8(11.4)	0.256	**0.001**	0.218
Psychoses	101(2.6)	98(3.9)	1(0.1)	2(1.7)	0(0)	0.238	**<.0001**	0.527
Hypertension	1261(32.3)	903(35.6)	311(26.2)	33(28.7)	14(20.0)	**0.044**	**<.0001**	0.224
Hepatitis C	519(13.3)	415(16.4)	80(6.8)	19(16.5)	5(7.1)	1.000	**<.0001**	0.074

*Note: P*-values computed using χ^2^ test for categorical variables and 2-sample t-test for continuous variables; *P*-values in bold denote statistical significance at the 0.05 level. ART = antiretroviral therapy. IQR = interquartile range; MSM = men who have sex with men. VL = Viral Load

^1^*P*-values refer to the χ^2^ or t-test between the ART-experienced and ART-naïve groups

^2^A total of 104 ART-experienced participants were excluded due to other/unknown insurance status

^3^A total of 11 ART-naive participants were excluded due to other/unknown insurance status^4^Year since HIV diagnosis computed as of June 30, 2015

^5^Other/unknown HIV risk defined as the sum of Blood Transfusion, Coagulation Disorder, Other, Perinatal, and Unknown categories

^6^Selected comorbidities based on top 5 most common conditions in the DC Cohort at study enrollment based on ICD-9/10 diagnosis data

### Laboratory monitoring

By IOM quality of care standards in unadjusted analysis, 82.8% of all PWH achieved regular CD4 monitoring, 83.9% achieved regular VL monitoring, and 72.2% achieved durable VS. In unadjusted analyses, among ART-naïve PWH, publicly-insured participants were significantly more likely than those with private insurance to meet the VL monitoring standard (83.5% vs 70.0%, *p* = 0.042) and were more likely to meet the CD4 monitoring standard, although not statistically significant (79.1% vs 65.7%, *p* = 0.057) (see Table 2). At community-based clinics, ART-naïve PWH with public insurance were more likely to meet the CD4 monitoring standard than privately-insured PWH (78.1% vs 41.2%, *p* = 0.006) yet no more likely for VL monitoring (82.2% vs 64.7%, *p* = 0.180).

**Table 2:** IOM standards of care among treatment-naive DC Cohort participants, by insurance type and clinic type in Washington, DC, 2011–2015

	Total N(%)	Regular CD4 monitoring^a^ N(%)	*P*-value	Regular VL monitoring^b^ N(%)	*P*-value	Durable viral suppression^c^ N(%)	*P*-value
**Insurance**							
Public	115(62.2)	91(79.1)	0.057	96(83.5)	**0.042**	61(53.1)	**0.030**
Private	70(37.8)	46(65.7)		49(70.0)		49(70.0)	
**Site**							
Hospital-based	95(51.4)	73(76.8)	0.405	74(77.9)	0.870	51(53.7)	0.134
Community-based	90(48.7)	64(71.1)		71(78.9)		59(65.6)	
Site*Insurance							
Hospital-based/ Public	42(44.2)	34(80.9)	0.468	36(85.7)	0.137	16(38.1)	**0.008**
Hospital-based/ Private	53(55.8)	39(73.6)		38(71.7)		35(66.1)	
Community-based/ Public	73(81.1)	57(78.1)	**0.006**	60(82.2)	0.183	45(61.6)	0.157
Community-based/ Private	17(18.9)	7(41.2)		11(64.7)		14(82.4)	

*Note*: ART status was determined at enrollment date. P-values based on X^2^ statistics and cross checked with Fisher’s exact test

^a^ Regular CD4 monitoring is defined as at least two CD4 lab measures 30 days apart in the 12 months following the index date

^b^ Regular VL monitoring is defined as at least two CD4 lab measures 30 days apart in the 12 months following the index date

^c^ Durable viral suppression is defined as last viral load < 50 copies/ML in the 12 months following the index date. Index date was defined as either the date of ART initiation or the date of study enrollment, whichever was later

Among ART-experienced PWH, those with public insurance were more likely to meet the CD4 monitoring standard (84.9% vs 79.7%, *p* < 0.0001) and VL monitoring standard (86.4% vs 79.5%, *p* < 0.0001) than those with private insurance (see Table 3). ART-experienced PWH at community clinics received more regular CD4 monitoring (84.9% vs 80.8%, *p* = 0.001) and VL monitoring (87.1% vs 80.1%, *p* < 0.001) than those at hospital clinics. ART-experienced publicly-insured PWH at hospital clinics are more likely to meet the CD4 monitoring standard (public 84.1% vs private 78.6%, *p* = 0.009) and the VL monitoring standard (83.1% public vs 78.1% private, *p* = 0.020).

**Table 3:** IOM standards of care among treatment-experienced DC Cohort participants, by insurance type and clinic type in Washington, DC, 2011–2015

	Total N(%)	Regular CD4 monitoring^a^ N(%)	*P*-value	Regular VL monitoring^b^ N(%)	*P*-value	Durable virologic suppression n^c^ N (%)	*P*-value
**Insurance**							
Public	2537(68.2)	2154(84.9)	**<.0001**	2191(86.4)	**<.0001**	1760(69.4)	**<.0001**
Private	1186(31.8)	945(79.7)		943(79.5)		951(80.2)	
**Site**							
Hospital-based	1513(40.6)	1222(80.8)	**0.001**	1211(80.1)	**<.0001**	1167(77.1)	**<.0001**
Community-based	2210(59.4)	1877(84.9)		1923(87.1)		1544(69.8)	
Site*insurance							
Hospital-based/Public	601(39.7)	505(84.1)	**0.009**	499(83.1)	**0.018**	412(68.6)	**<.0001**
Hospital-based/Private	912(60.3)	717(78.6)		712(78.1)		755(82.8)	
Community-based/Public	1936(87.6)	1649(85.2)	0.417	1962(87.4)	0.151	1348(69.6)	0.574
Community-based/Private	274(12.4)	228(83.2)		231(84.3)		196(71.5)	

*Note*: ART status was based on treatment status at study enrollment. *P*-values based on X^2^

^a^Regular CD4 monitoring is defined as at least two CD4 lab measures 30 days apart in the 12 months following the index date

^b^Regular VL monitoring is defined as at least two CD4 lab measures 30 days apart in the 12 months following the index date

^c^Durable viral suppression is defined as last viral load < 50 copies/ML in the 12 months following the index date. Index date was defined as either the date of ART initiation or the date of study enrollment, whichever was the most recent

### HIV outcomes

In unadjusted analysis, privately-insured ART-naïve PWH were less likely to meet the CD4 and VL monitoring standards but more likely to experience durable VS than those with public insurance (70.0% vs 53.1%, *p* = 0.030) (see Table 2). Among ART-naïve PWH, durable VS did not differ by clinic type (hospital 53.7% vs community 65.6%, *p* = 0.134). However, at hospital clinics, ART-naïve privately-insured PWH were significantly more likely to achieve durable VS than publicly-insured PWH (66.1% vs 38.1%, *p* = 0.008). ART-naïve PWH with public insurance at community clinics had a significantly longer period from HIV diagnosis to ART initiation than those at hospital clinics (85.1 vs 31.3 months, *p* = 0.0001).

Among ART-experienced PWH, although publicly-insured PWH were more likely to meet CD4 and VL monitoring standards, they were less likely than private-insured PWH to have durable VS (69.4% vs 80.2%, *p* < 0.0001). Similarly, ART-experienced PWH at community clinics received more regular CD4 and VL monitoring but were less likely to achieve durable VS than those at hospital clinics (hospital 77.1% vs community 69.8%, p < 0.0001) (see Table 3). This finding is especially pronounced at hospital clinics when including insurance in the analysis: despite being more likely to meet CD4 and VL monitoring standards, ART-experienced publicly-insured PWH at hospital clinics are less likely to achieve durable VS than privately-insured PWH (68.6% vs 82.8% p < 0.0001).

### Multivariate analysis

Multivariate regression modeling adjusting for demographic characteristics, years since HIV diagnosis, HIV risk categories, and AIDS diagnosis among ART-experienced PWH showed no significant difference in durable VS by insurance type or by site (see Table 4). However, in the adjusted model, among those attending hospital clinics, privately-insured PWH were more likely to have durable VS compared with publicly-insured PWH (AOR = 1.59, 95% CI: 1.20, 2.12; *p* = 0.001).

**Table 4:** Adjusted odds ratio (aOR) on factors associated with durable HIV virologic suppression among DC Cohort participants in Washington, DC, 2011–2015

Factors	Level	Reference^a^	aOR^b^	Lower 95% CI	Upper 95% CI	*P*-value
Insurance	Private	Public	1.02	0.76	1.37	0.908
Site of care	Hospital	Community	0.83	0.67	1.03	0.089
Insurance * Site	Privately-Insured in Hospital	Publicly-Insured in Hospital	1.59	1.20	2.12	**0.001**
	Hospital with Privately-Insured	Community with Privately-Insured	1.31	0.92	1.85	0.138
Employment status	Unemployed	Employed	0.77	0.61	0.98	**0.033**
	Other/Unknown	Employed	0.68	0.54	0.85	**0.001**
Age(at time of enrollment)	Age^c^	Ten year increment	1.30	1.21	1.38	**<.0001**
Race/Ethnicity^d^	Hispanic	NH Black	2.46	1.66	3.65	**<.0001**
	NH White	NH Black	1.49	1.13	1.95	**0.004**
	Other/Unknown	NH Black	1.41	0.93	2.13	0.105
AIDS diagnosis	Yes	No	0.61	0.52	0.71	**<.0001**
Years since enrollment	Year	One year increment	1.55	1.46	1.64	**<.0001**

Note: P-values based on X^2^. CI = confidence interval. aOR = adjusted odds ratio

^a^Reference level for categorical variable is the group with largest number of PWH

^b^ Multivariate regression modeling adjusted for demographic characteristics (age, gender, race/ethnicity, housing and employment status), years since HIV diagnosis, HIV risk categories, and AIDS diagnosis

^c^Patient age is divided by 10, such that one unit increase in age represents the effect of 10 years

^d^Race/ethnicity was forced into the model that was selected by backward elimination procedure and cross checked with forward selection model selection process

For every 10 years in age, the odds of achieving durable VS increased by almost 30% (AOR 1.30, CI 1.21–1.38, *p* < .0001). We found that both non-Hispanic (NH) whites and Hispanics had an increased likelihood of durable VS compared to NH blacks (NH whites: AOR 1.46; CI 1.13–1.95, *p* = 0.043; Hispanics: AOR 2.46, CI 1.66–3.65, *p* < 0.001). Durable VS was inversely associated with having an AIDS diagnosis. Neither housing status nor mode of HIV transmission was significant.

## Discussion

In Washington, DC, early Medicaid expansion and a well-funded ADAP have provided broad healthcare access with potential to improve HIV care outcomes. However, this post-ACA analysis shows important disparities in the rates of HIV laboratory monitoring and durable VS in publicly- and privately-insured PWH in Washington, DC. Despite 72% overall prevalence of durable VS among DC cohort participants with stable insurance, inequities in HIV outcomes clearly exist. Among both ART-naïve and -experienced PWH in unadjusted analysis, those with public insurance were more likely to meet IOM VL and CD4 monitoring standards yet less likely to experience durable VS than those with private insurance, as suggested by an earlier Cohort analysis [9] . Although the individual effects of site and insurance did not persist in adjusted analyses, the site-insurance interaction effect persisted, with privately-insured PWH receiving care in hospital clinics significantly more likely than publicly-insured PWH to achieve durable VS. This is significant in light of a previous Cohort study showing PWH at hospital clinics were less likely to be retained in care than those at community clinics [11].

Frequent lab monitoring alone is not sufficient to produce VS among vulnerable PWH, as many factors that determine successful medication adherence occur outside of the clinic context. The significantly higher burden of medical and mental health comorbidities faced by publicly-insured PWH may affect their ability to adhere with HIV medications due to pill burden, medication side-effects, or drug-drug interactions. Mental health and substance use comorbidities are additional well-established barriers to medication adherence [15–17]. Despite improved access to government-sponsored health insurance, publicly-insured PWH also face structural barriers to health care such as medication copays (a one dollar copay for each DC Medicaid prescription can be a significant financial obstacle for some), decreased health literacy, difficulty renewing public insurance in a timely manner to prevent medication discontinuation, and lack of transportation to pharmacies for medication retrieval [18, 19].

Clinical indicators endorsed by the IOM are a one-size-fits-all approach and do not account for insurance status, which may act as a direct marker of socioeconomic status [20]. Consistent with our findings, a study from New York found PWH with fewer economic barriers had more effective self-management and required fewer clinical visits to achieve VS [21]. Our results suggest that PWH with public insurance may require a broader set of wrap-around interventions than frequent laboratory monitoring to achieve VS; possibilities include transportation assistance, medication adherence assistance, elimination of medication copays for Medicaid recipients, less frequent mandatory renewals of public insurance, home- or community-based medical visits, improved mental health and substance use care linkage, and increased availability of mental health and addiction services.

At hospital clinics in the DC Cohort, publicly-insured PWH were less likely than privately-insured PWH to achieve VS, despite increased lab monitoring. These results suggest that frequency of HIV monitoring should be tailored not only to an individual’s socioeconomic indicators for which insurance may be a proxy, but also to clinic type. Within the DC Cohort, clinics with Patient-Centered Medical Home components and programs actively monitoring retention and ART adherence have faster time to ART initiation and VS [22]. Further research is needed to identify if a clinic’s ability to effectively address structural barriers to health care is improved by co-located services such as on-site mental health providers, medical case management (MCM), addiction services, dental services, nutritionists, transportation assistance, legal services, insurance navigation, and “Red Carpet” services. MCM, for example, has been shown to improve retention in care but not VS among PWH in Washington, DC [23].

“Red Carpet” programs (enacted in 4 of 8 hospital clinics and 4 of 5 community clinics in the Cohort between 2008 and 2012) offer immediate, post-HIV diagnosis linkage to public benefits, insurance navigation, medical case management and HIV specialty medical care. Through these programs, community clinics may contribute to reducing disparities in durable VS among publicly- and privately-insured PWH. Further analysis of “Red Carpet” program availability and impact is needed.

Our findings are consistent with another recent DC Cohort analysis showing that PWH who were retained in care were more likely to receive care at community clinics, while those with VS were less likely to receive care at community clinics [24]. Geographic clusters of DC Cohort participants within Washington, DC with low retention in care but high VS were located in more affluent areas of Washington, DC. Thus, retention may relate to but not fully predict VS. Retention measures based on visit or lab frequency may misclassify PWH suppressed on ART as not retained [25].

Unlike other large HIV clinical cohorts which capture mainly MSM or heterosexual PWH, the DC Cohort represents a large urban cohort with diverse race/ethnicity, insurance type, and HIV exposure risk (38% MSM; 34% heterosexual participants). This diverse sampling permits a real-world look at the wide variety of factors which influence HIV clinical outcomes in addition to insurance type. Our sample diversity increases our confidence that the effects on HIV outcomes on insurance type and clinic site are generalizable across diverse groups of PWH. It is of interest that our publicly-insured patients tended to be more female, black, heterosexual, unemployed, and receiving care at community clinics. Further correlative analyses probing more deeply into these differences are ongoing within the DC Cohort. The diversity of our clinics serving PWH permits evaluation of specific opportunities to improve outcomes for individuals of a particular risk group and simultaneously decrease barriers to care that are similar for all people with HIV.

Several limitations should be considered. Because the DC Cohort does not provide pre-ACA data, we instead analyzed by insurance type in the post-ACA period. The cohort database lacks pharmacy data for medication adherence assessments as well as data on site-level services that impact care delivery; future analyses should include adherence to drug refills as a predictor for virologic suppression. Because the cohort database was not designed to track individual changes in insurance over time, we cannot account for underlying characteristics of Medicaid enrollees as Medicaid enrollment criteria changed.

Excluding PWH with changes to insurance status during the study period potentially removed PWH with disruptive personal circumstances and perhaps suboptimal virologic outcomes; however, PWH who cycle in and out of HIV care with stable insurance were included in our analysis. Incomplete data for employment and housing status and lack of income data reduced the ability to evaluate the impact of these socioeconomic factors on HIV outcomes. We were unable to perform multivariate interaction analyses on our data among ART-naïve PWH due to small sample size. Secondary insurance type was collected at baseline, but not analyzed.

## Conclusion

The DC DOH aims for 90% of PWH on ART to achieve VS by 2020; to meet this goal, we must continue to improve outcomes for the most vulnerable PWH [26]. Publicly insured PWH receiving care in hospital clinics have worse virologic outcomes than their privately insured peers, and additional work is needed to address this disparity. Our data demonstrate that IOM standards for laboratory monitoring do not predict virologic success in diverse populations in Washington, DC. Guidelines for quality of care need to be reconsidered to better serve at-risk populations.

## Data Availability

The datasets used and/or analysed during the current study are available from the corresponding author on reasonable request.

## Abbreviations

ACA: Affordable Care Act
ADAP: AIDS Drug Assistance Program
ART: antiretroviral therapy
ARV: antriretroviral
DC: District of Columbia
IOM: Institute of Medicine
MCM: medical case management
MSM: men who have sex with men
NH: non-Hispanic
PWH: people with HIV
VS: virologic suppression
IRB: Institutional Review Board
